# Tumor glycolysis as a target for cancer therapy: progress and prospects

**DOI:** 10.1186/1476-4598-12-152

**Published:** 2013-12-03

**Authors:** Shanmugasundaram Ganapathy-Kanniappan, Jean-Francois H Geschwind

**Affiliations:** 1 Russell H Morgan Department of Radiology & Radiological Sciences, Division of Interventional Radiology, Johns Hopkins University School of Medicine, 600 N. Wolfe Street, Blalock Building 340, 21287 Baltimore, MD, USA; 2 Russell H. Morgan Department of Radiology and Radiological Sciences, Division of Vascular and Interventional Radiology, Sheikh Zayed Tower, Suite 7203, The Johns Hopkins Hospital, 1800 Orleans Street, 21287 Baltimore, MD, USA

**Keywords:** Glycolysis, Antiglycolytic agents, Cancer metabolism, Chemotherapy

## Abstract

Altered energy metabolism is a biochemical fingerprint of cancer cells that represents one of the “hallmarks of cancer”. This metabolic phenotype is characterized by preferential dependence on glycolysis (the process of conversion of glucose into pyruvate followed by lactate production) for energy production in an oxygen-independent manner. Although glycolysis is less efficient than oxidative phosphorylation in the net yield of adenosine triphosphate (ATP), cancer cells adapt to this mathematical disadvantage by increased glucose up-take, which in turn facilitates a higher rate of glycolysis. Apart from providing cellular energy, the metabolic intermediates of glycolysis also play a pivotal role in macromolecular biosynthesis, thus conferring selective advantage to cancer cells under diminished nutrient supply. Accumulating data also indicate that intracellular ATP is a critical determinant of chemoresistance. Under hypoxic conditions where glycolysis remains the predominant energy producing pathway sensitizing cancer cells would require intracellular depletion of ATP by inhibition of glycolysis. Together, the oncogenic regulation of glycolysis and multifaceted roles of glycolytic components underscore the biological significance of tumor glycolysis. Thus targeting glycolysis remains attractive for therapeutic intervention. Several preclinical investigations have indeed demonstrated the effectiveness of this therapeutic approach thereby supporting its scientific rationale. Recent reviews have provided a wealth of information on the biochemical targets of glycolysis and their inhibitors. The objective of this review is to present the most recent research on the cancer-specific role of glycolytic enzymes including their non-glycolytic functions in order to explore the potential for therapeutic opportunities. Further, we discuss the translational potential of emerging drug candidates in light of technical advances in treatment modalities such as image-guided targeted delivery of cancer therapeutics.

## Introduction

Glucose metabolism in cancer cells is primarily characterized by two major biochemical events: (i) increased glucose uptake and (ii) aerobic glycolysis, the process of conversion of glucose into pyruvate eventually resulting in the production of lactate (fermentation). The former has already been exploited clinically to diagnose cancer and assess tumor response through the utilization of radiolabeled glucose analog, ^18^Fluoro-deoxyglucose (FDG) in positron emission tomography (PET). PET imaging, combined with computed tomography (CT), plays an indispensable role in modern diagnostic oncology [1]. But it is the notion that tumor glycolysis could be used as a potential target for therapy that remain the most intriguing. The existence of a link between aerobic glycolysis (i.e. glycolysis in the presence of oxygen) and tumorigenesis has been known for several decades ever since the German scientist Otto Warburg proposed the “Warburg hypothesis” known as the “Warburg effect” [2,3]. Yet, the underlying mechanistic details pertinent to the causes and consequences of such metabolic phenotype remained unclear. Conceptual advances in the past decades have improved our understanding on the biological significance of tumor metabolism [4]. As a result, deregulated or altered energy metabolism has been recognized as one of the “hallmarks of cancer” [5].

It is increasingly evident that oncogenes and tumor suppressors regulate altered energy metabolism. Oncogenic mutations culminate in the up-regulation of glucose transporters (e.g. GLUT 1, GLUT 3) [6,7] thus facilitating increased glucose consumption by cancer cells, which in turn increases the rate of glucose metabolism. Conversely, the glycolytic/metabolic phenotype confers selective advantage to cancer cells by supporting uninterrupted growth. For example, a higher glycolytic rate in tumor cells has been shown to promote resistance to chemotherapeutics. In the cervical cancer cell line, HeLa for example, the enzyme pyruvate dehydrogenase kinase (PDK) isoforms PDK1 and PDK3 have been demonstrated to provide resistance to chemotherapeutics [8]. Similarly, in the colon carcinoma cell line, LoVo it has been demonstrated that increased aerobic lactate production (glycolysis) correlated with drug resistance [9]. Thus, interrupting or possibly disrupting tumor glycolysis will impact tumor growth by energy depletion as well as sensitization to therapeutics especially, in light of the recent reports that have elucidated cancer-specific advantages of aerobic glycolysis [10-13]. Several authors have delineated a wealth of information on the biochemical targets of glycolysis and their potent antagonists or inhibitors with promising anticancer effects (refer reviews [14-18]). Our goal in this review is to discuss the cancer-specific intricacies and advantages of glycolysis in the light of recent research underscoring the clinical relevance of targeting it for cancer therapy.

### Glycolysis in cancer

The fact that cancer cells express the glycolytic phenotype has long been known (refer review, [19]). However, until recently, the dependence on such a phenotype remained unclear. In an elegant report, Bonnet et al. [20] demonstrated that reversing the glycolytic phenotype to oxidative phosphorylation (OXPHOS) in cancer cells resulted in the induction of cell death. Further, when the mitochon-drial-K^+^ channel axis of cancer cells is suppressed, a mere restoration of mitochondrial-K^+^ channel function is sufficient to promote apoptosis. This report supports two major hypotheses, (i) reversal of the glycolytic phenotype to oxidative phosphorylation can promote cancer cell death and (ii) glycolysis can facilitate tumor growth despite a suppressed mitochondria-K^+^ channel axis.

Understandably, the metabolic switch from mitochondrial respiration to glycolysis during hypoxia (where oxidative phosphorylation will be inactive) as well as mitochondrial dysfunction [21,22] are critical for cancer cell growth. Yet, the presence of aerobic glycolysis under normoxic conditions in the context of functionally efficient mitochondria is also very intriguing. Mitochondrial impairment or defective oxidative phosphorylation is frequently found in cancer. It is known that mutations in mitochondrial DNA (mtDNA) affect the enzymes involved in OXPHOS, at least three enzymes from the TCA cycle, succinate dehydrogenase (SDH), fumarate dehydrogenase (FDH) and isocitrate dehydrogenase (IDH) (reviewed by Wallace [23]) whereas mitochondrial gene mutations in the nuclear DNA (nDNA) primarily affect the bioenergetics status of cancer cells (reviewed by Wallace [23]). These enzymatic mutations have been linked to several intrinsic pathways that together or independently can reprogram the metabolic circuitry of cancer cells. For example, SDH mutation results in the accumulation of succinate which in turn inhibits prolyl hydroxylase dehydroganse (PHD) eventually contributing for the stabilization of HIF-1α. This mechanism is sufficient to recognize the importance of HIF-1α’s role as an activator of aerobic glycolysis and lactate production. Thus it is clear that mitochondrial defect in cancer cells can cause a shift in energy metabolism.

On the other hand, cancer cells subjected to mtDNA gene mutations or deletions show reduced colony formation, growth rate and diminished tumorigenicity [24]. Based on this and similar reports, if impaired mitochondria were truly a “common cause of cancer growth” as proposed by Warburg, then it is difficult to explain the rapid proliferation, formation of metastases and chemoresistance typical of cancer cells. It could then be that such cancer cells harbor the functionally normal mitochondria from surrounding normal cells [25]. If this were the case, it could not support the theory that a mitochondrial defect is at the origin of “aerobic glycolysis or lactate production” as normal mitochondria (located in adjacent normal healthy cells) could compensate for the OXPHOS function. Nevertheless, a wealth of data indicate that a link between mitochondrial function and cancer progression exists, especially with the energy metabolism of cancer cells, although a distinctive step-wise mechanistic principle underlying the origin of cancer remains extremely controversial.

Recent investigations have shed light on the understanding of the benefits and selective advantages of aerobic glycolysis. Although glycolysis yields a lower amount of ATP compared to mitochondrial OXPHOS, several key benefits inherent in aerobic glycolysis drive cancer cells to favor glycolysis over mitochondrial oxidation [26]. First, the rate of glycolysis and turnover of glucose into lactic acid is accelerated thereby resulting in faster and greater ATP production. Pfeiffer et al. [27] have postulated that the high-rate but low yield ATP producing pathway (glycolysis) confer selective advantage under competition for shared energy sources, adding an evolutionary significance to glycolysis [28]. The rate of ATP production may be 100 times faster with glycolysis than with OXPHOS [29]. The low yield of ATP with glycolysis is however sufficient to meet intracellular demand. Rapidly dividing cells such as microorganisms (with a doubling time ranging from a few minutes to several hours) require ATP for proliferation whereas cancer cells with a comparatively longer doubling time (days rather than minutes) may require ATP primarily only for cell maintenance (rather than for proliferation). For all these reasons, the ATP formed through glycolysis is sufficient for cancer growth. It is therefore likely that the increased rate of ATP production resulting from glycolysis confers a selective growth advantage to cancer cells [30,31]. Second, in addition to ATP, cancer cells require further metabolic intermediates and precursors that are critical for the biosynthesis of macromolecules, the ultimate building blocks indispensable to increase the tumor mass during growth and proliferation [32]. The accumulation of glycolytic intermediates is known to promote the pentose phosphate pathway PPP resulting in the generation of NADPH and ribose-5-phosphate. Both, NADPH and ribose-5-phosphate are essential for the biosynthesis of lipids and nucleic acids. Lastly, the production of NADPH enables the cancer cells to maintain adequate levels of reduced forms of glutathione (GSH), a key non-enzymatic antioxidant. GSH plays a pivotal role in protecting cancer cells against antineoplastic agents by maintaining the redox status as well as by counteracting some of the effects from chemotherapeutic agents (reviewed [33,34]). In this context, under experimental conditions, Zhou et al. [28], have demonstrated that chemoresistant cell lines have elevated aerobic glycolysis indicating a biochemical link between resistance and glycolysis. Apart from the resistance to chemotherapeutics, aerobic glycolysis has also been implicated in resistance to radiotherapy. Indeed, Pitroda et al. [35] have demonstrated that regulation of glycolytic or energy metabolic pathway affects the sensitivity of tumor cells.

The PPP plays a pivotal role in macromolecular biosynthesis. Recent evidence indicates that it also contributes to therapeutic resistance as an antioxidant system to chemo- and radiation therapies [36]. Among several enzymes involved in the PPP, the transketolase (TKTL1) has gained increased attention owing to its involvement in cell survival under stress or starvation [37-39]. Other data also indicate that TKTL1 affects the chemosensitivity of cancer cells to drugs such as imatinib [40], cetuximab [41]. Thus it is evident that aerobic glycolysis in conjunction with the pentose shunt pathway provide multiple benefits to cancer cells such as promoting tumor progression and providing resistance to therapy. Hence, this key signature of cancer cells, tumor metabolism, particularly the tumor glycolysis, provides an ideal target for therapeutic intervention.

### Non-glycolytic functions of glycolytic enzymes and the metabolic intermediates

**Figure 1:**
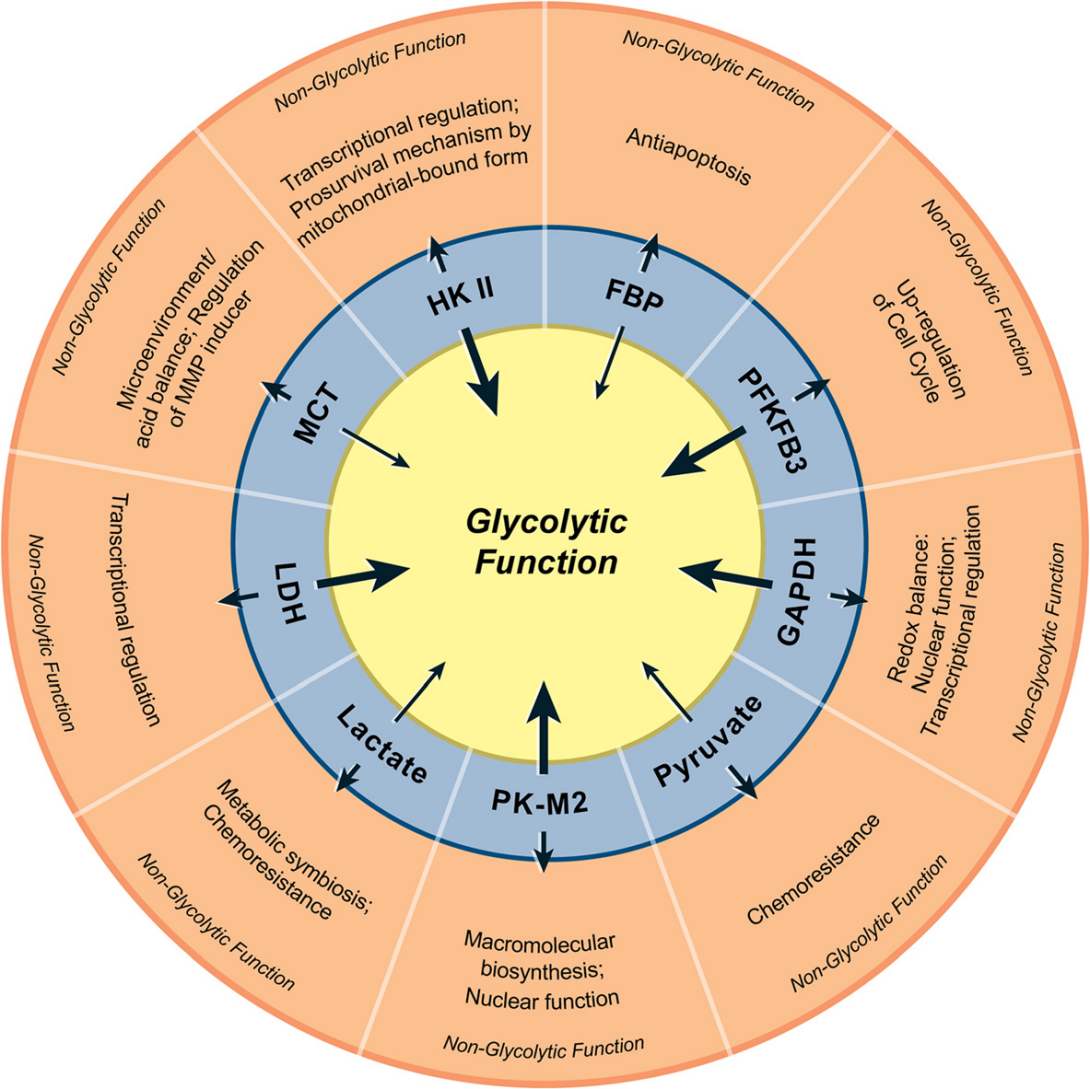
**Non-glycolytic functions of glycolytic enzymes and metabolic intermediates.** In the innermost circle, thick arrows represent enzymes and thin arrows indicate intermediate metabolites. The short arrows pointing towards the outer circle represent the non-glycolytic functions of corresponding enzymes/metabolites.

Many enzymes of the glycolytic pathway also play significant roles in several non-glycolytic processes that enable the cancer cells to meet other cellular demands. As shown in Figure 1, enzymes such as hexokinase II (HKII), glyceraldehyde-3-phosphate dehydrogenase (GAPDH), pyruvate kinase (PK)-M2 isoform and lactate dehydrogenase (LDH) are known to be involved in a number of subcellular functions including transcriptional regulation and phosphorylation of histones [42].

For example, the mitochondrial membrane-bound HKII antagonizes the proapoptotic machinery there by provides survival advantage to cancer cells [43,44]. In addition, HKII is also involved in transcriptional regulation, a functional property characteristic of nuclear proteins [42]. Similarly, GAPDH plays a crucial role in the maintenance of cellular redox balance as it catalyses the first step to produce NADH (extensively reviewed by Seidler [45]). It is also known that GAPDH plays a pivotal role in protecting the cells from free radical or ROS-mediated injury. As for the nuclear functions of GAPDH available reports indicate that GAPDH might be involved in both pro-apoptotic and oncogenic processes (Seidler, [46]). Its oncogenic role involves the indirect participation in nucleic acid binding properties of hepatitis viruses – a function that correlates with liver carcinogenesis [47]. What remains to be elucidated is the identification of the intracellular mechanism that directs the proapoptotic or oncogenic role of GAPDH. Next, PK-M2 is involved in the regulation of macromolecular biosynthesis (e.g. nucleic acid) [48] and multiple reports have established its role in tumor progression [49-51]. In addition to its involvement in biosynthesis, nuclear translocation of its phosphorylated form has been shown to promote the Warburg effect [52]. Several reports have indicated that PK-M2 strongly participates in diverse non-glycolytic functions [53]. PK-M2 acts as a kinase and phosphorylates histone H3 to favor tumorigenesis [54], as a nuclear protein it transactivates β-catenin [55], and as a phosphotyrosine binding protein it interacts with other proteins as well [56]. Evidence from gene silencing experiments demonstrate that the enzyme, LDH cooperates with Oct-4, a transcriptional factor, during gastric tumorigenesis. Silencing LDH abrogated tumorigenicity by Oct-4 down regulation (19). Thus many glycolytic enzymes participate or influence several non-metabolic functions.

As with glycolytic enzymes, some of the metabolic intermediates of glycolysis have also been associated with non-glycolytic pathways. For example, Fructose-1, 6 bisphosphate plays an anti-apoptotic role in cancer cells by maintaining the cytochrome C in a reduced, non-active state [57]. Similarly, pyruvate contributes to chemoresistance by over-expressing the p-glycoprotein [58]. The export and import of lactate (the product of pyruvate oxidation) is achieved through the transporters known as monocarboxylate transporters (MCTs). The non-glycolytic role of MCTs include the regulation of the CD147, a matrix metalloproteinase inducer, which increases the invasion and metastatic potential of cancer cells [59,60]. Collectively, these findings strongly suggest that many of the enzymes and metabolic intermediates of tumor glycolysis also play a key role beyond glycolysis, thereby facilitating the growth and survival of cancer cells.

**Figure 2:**
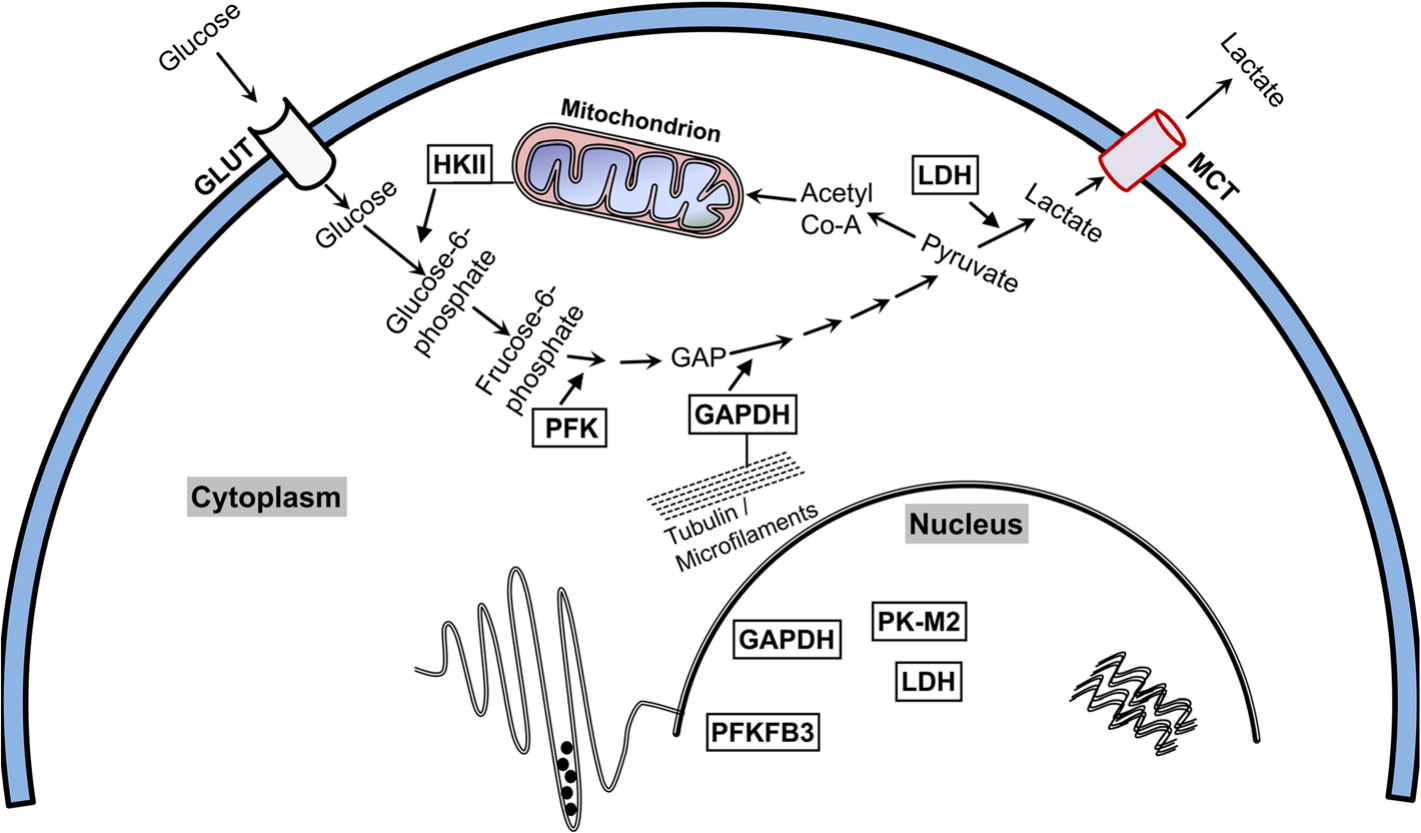
**Schematic showing the distribution of glycolytic enzymes in various subcellular compartments.** HKII is a cytoplasmic enzyme however its localization to mitochondrial membrane has been established. As for binding with tubulin or actin filaments existing data are strong enough to include only GAPDH and for other glycolytic enzymes it remains to be known. The nuclear translocation has been well documented for the enzymes GAPDH, LDH, PFKFB3 and PK-M2.

Increasing evidences demonstrate that glycolytic enzymes translocate to different subcellular compartments where they can interact with subcellular structures to an appreciable degree resulting in significant differences in their primary and secondary functions (Figure 2). The enzyme HKII which catalyses the first rate-limiting step of glucose metabolism is located proximally to the mitochondria in order to facilitate the immediate utilization of ATP. Similarly, other glycolytic enzymes such as GAPDH and aldolase are anchored by actin filament-like structures located in close proximity to the segments of glycolysis taking place in the cytoplasm [61].

Whereas the necessity and significance of the association between glycolytic enzymes and cytoskeletal structures remains obscure, the nuclear functions of glycolytic enzymes are sufficiently documented. Indeed, one of the isoforms of phosphofructo kinase (PFK), the PFKFB3 impacts cancer cell proliferation by its nuclear translocation [62]. Similarly, the direct binding of GAPDH to telomeric DNA protects telomeres against chemotherapy-induced rapid degradation [63]. GAPDH also enhances the transcriptional activity of androgen receptors in prostate cancer cells [64]. Finally, the nuclear translocation of LDH modulates the functions of DNA polymerases alpha, delta and epsilon [65]. It is convincingly evident that glycolytic enzymes participate in several non-glycolytic processes at various subcellular locations including the mitochondrial-membrane, as well as nuclear and cytoplasmic compartments.

### Relevance for targeting glycolysis

A higher lactate level significantly correlates with tumor recurrence and the metastatic potential of tumors resulting in poor patient outcomes [66]. As lactate level indicates the prevalence of glycolytic phenotype targeting such tumors through antiglycolytic agents likely to be very effective. Lactate was originally thought to be an acidic molecule that must be exported from cancer cells to prevent deleterious intracellular acidification. Recently, different roles of lactate export/import have been implicated with some directly contributing to cancer survival and growth and others to the metabolism of normoxic cancer cells that do not produce or excrete lactic acid. In this way, a sort of “metabolic symbiosis” exists within the tumor [67]; the lactate produced and extruded by hyperglycolytic or hypoxic cancer cells is able to re-enter normoxic cancer cells and be utilized to generate energy through mitochondrial oxidation [68]. In the heterogeneous tumor microenvironment characteristic of many solid tumors where both normoxic and hypoxic conditions co-exist, (depending upon angiogenesis and their proximity to blood vessels), the described “give and take lactate” mechanism [69] would mutually benefit both the lactate-exporting cells and the surrounding lactate-importing cells. In addition, the presence of lactate within the tumor microenvironment, which causes extreme acidic conditions, enables the deactivation or even inactivation of several chemotherapeutic agents. This process of lactate export and import is achievable by the over-expression of MCTs (primarily the MCT1 and MCT4). Note that the MCTs are over expressed in most tumors [70]. The release of lactate occurs through MCT4, whereas its uptake occurs through MCT1 [71]. In mouse and human tumors, MCT1 was found to be the major transporter ensuring lactate uptake by oxidative tumor cells and MCT4 as a hypoxia-induced transporter involved in the removal of lactate from glycolytic cells. Interestingly, MCT1 was found in the tumor cells of vascularized area whereas MCT4 was consistently concentrated in hypoxic regions correlating well with their known respective functions.

Recently, using untransformed primary breast cells (HMEC) as controls, Hussien and Brooks [72] demonstrated that a significant correlation exists between the expression profile of MCT isoforms (MCT-1 and 4) and the abundance of LDH isoforms (LDH A and B) in breast cancer cell lines (MDA-MB231 and MCF-7). In the MCF-7 cell line MCT1 (export of lactate) is abundant and LDHA, which converts pyruvate to lactate, is upregulated. On the other hand in the MDA-MB-231 cells, MCT4 is over expressed (uptake of lactate to be converted back to pyruvate for utilization in TCA cycle), LDHB is abundant. Thus cancer cells organize their glycolytic phenotype in a programmed fashion in order to achieve maximum efficiency. Thus it is conceivable that inhibition of glycolysis could be effective in killing both glycolytic and “symbiotic” non-glycolytic tumor cells.

Multiple lines of evidences have established that a higher expression levels of GLUTs and of certain enzymes such as HKII, GAPDH, LDH and PFK-B is linked to malignant growth [73-75]. As discussed elsewhere, it is increasingly evident that the cancer specific up-regulation of glycolysis is regulated through oncogenes (e.g. c-myc, Akt). The oncogenic activation directly up-regulates glycolytic enzymes [76] and/or through the hypoxia induced HIF-1alpha activation, which is a characteristic of tumor microenvironment [77]. The later has been experimentally verified using 3D-*in vitro* models, where spheroid-formation resulted in the promotion of a central hypoxic area eventually leading to an increase in the glycolytic flux [78]. Akt, the serine/threonine kinase, is an oncogene that promotes cancer growth [79]. Akt activates aerobic glycolysis, importantly, renders cancer cells dependent on glycolysis for survival [80].

Coordinated networks involving signaling pathways enable cancer cells to detect and integrate the immediate environmental conditions to balance their anabolic and catabolic processes. The mammalian Target of Rapamycin (mTOR) represents such a pathway where the intracellular energy sensing molecule AMPK can impact the mTOR complex I (mTORC1) mechanism of activation to either delay or halt the energy consuming synthetic processes [81]. Such an adaptation involves mTORC1-mediated regulation of the expression of glycolytic enzymes through the activation of genes such as c-myc and HIF1-alpha [81-83]. In summary, as aerobic glycolysis plays a major role in molecular events associated with oncogenesis targeting it could be not only a relevant but also a viable anticancer strategy.

### Molecular targets and inhibitors of glycolysis

**Figure 3:**
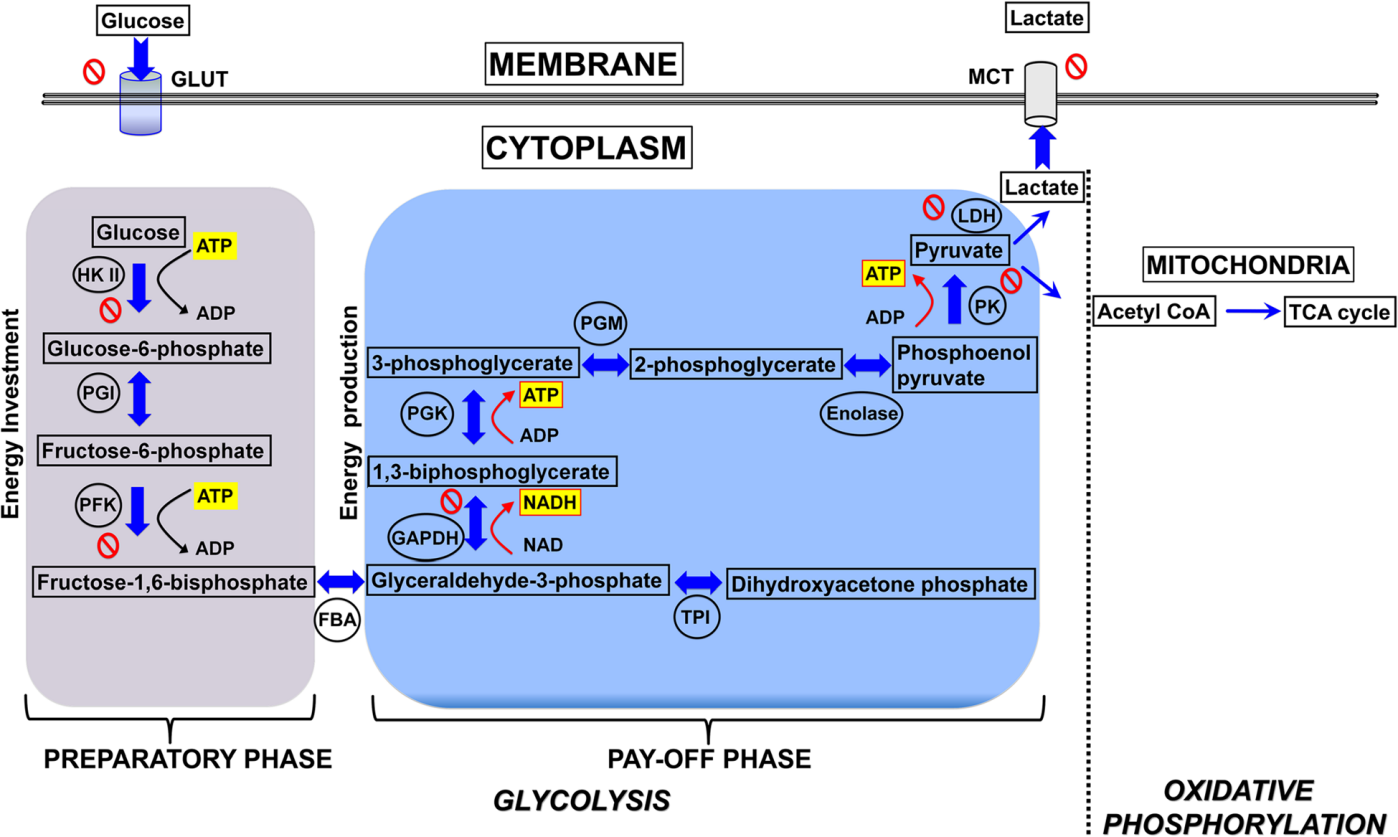
**Diagram showing the two phases of glycolysis and the molecular targets currently exploited for potential therapeutic drug strategies.** Energy molecules such as ATP and NADH are highlighted in yellow, black arrows indicate consumption while red arrows indicate the energy release. The enzymes involved in respective reactions are abbreviated and encircled, where as the block symbol shows the targets exploited for drug development in preclinical investigations.

Figure 3 depicts major biochemical reactions of glycolysis along with the enzymes involved and the energy utilized or produced during the process with an emphasis on current molecular targets. The most important role of glycolysis is to consume glucose and convert it into energy in the form of ATP. The consumption of glucose is an active process, which relies on specific transporters known as GLUTs. These GLUTs are over-expressed in almost all cancer types and hence contribute to the increased glucose utilization that is characteristic of the glycolytic phenotype, a key signature of cancer. The entire process of glycolysis can be divided between a “preparatory phase” where energy is consumed and a “pay-off phase” where net energy is generated in the form of ATP and NADH.

There are several approaches to disrupting glycolysis. Since cancer cells depend on increased utilization of glucose as compared to normal healthy cells, glucose deprivation could be an effective anticancer approach and possibly used as a cancer-preventive strategy. Indeed, carbohydrate-restricted diets to treat cancer patients have been reported to have therapeutic benefits [84].

An obvious direct approach would be to block the GLUTs, which would prevent glucose entry into the cancer cell and lead to total disruption of the glycolytic pathway. Several such compounds (e.g. Phloretin, WZB117, Fasentin) demonstrated anticancer effects in preclinical models [6,85]. However, selective blockade of GLUTs in tumor cells remains a critical challenge as GLUTs are ubiquitously expressed in all mammalian cells.

Another approach is to target the enzyme HKII that is responsible for the first step of glycolysis that converts glucose to glucose-6-phosphate. This enzyme plays a pivotal role in tumor glycolysis. First, it is a rate limiting step that provides direct feedback inhibition thereby preventing the consumption of cellular ATP in turn preserving precious energy within the cancer cell. Second, it has a low Km (high affinity) for glucose. This characteristic facilitates the initiation of glycolysis specifically in times of low serum glucose levels, and along with its subcellular localization, (bound to mitochondrial membrane) plays a pivotal role in the energy metabolism of cancer cells [86,87]. Lonidamine is an inhibitor of HKII and has completed phase III trial. However its clinical success has so far been impaired by significant pancreatic and hepatic toxicities [88]. Similarly, another glucose analog 2-deoxyglucose (2-DG) also showed promising anticancer effects in preclinical models [89]. However, later studies revealed that the principal mechanism underlying 2-DG’s anticancer effects vary [90,91]. Moreover, contrary to its widely believed anticancer effects, 2-DG was shown to activate pro-survival pathways in cancer cells [92]. In addition, hypoxic cells demonstrated chemoresistance against 2-DG [93]. Thus the success of 2-DG as a single agent for antiglycolytic therapy has been challenged. However, in combination treatments, 2-DG showed encouraging outcomes providing a new window of opportunity in combination therapy [94,95].

PFK catalyzes another rate-limiting step of glycolysis and is regulated by allosteric effectors and covalent modifications such as phosphorylation. It is activated by AMP and fructose 2,6-bisphosphate (F-2, 6-BP). An abundance of ATP inhibits the activity of PFK, presumably representing a regulatory mechanism. However, F-2, 6-BP has the capability to override the inhibitory effect of ATP, and to perpetuate uninterrupted glycolytic flux. Predictably, F-2, 6-BP is elevated in cancer cells [96]. It is regulated by the activity of a family of bi-functional enzymes including PFKFBs which is also up-regulated in cancer cells. As a result, specific inhibitors of PFKFB3 are being developed in several laboratories. Preliminary studies revealed promising anticancer effects [97] but further investigations are necessary to assess whether this approach could potentially be successful in the clinic.

An alternative promising therapeutic approach to date in terms of inhibiting tumor glycolysis has been targeting the enzyme GAPDH. In many ways the GAPDH reaction is unique because GAPDH catalyzes the very first step in which energy in the form of NADH is produced, the so-called “pay-off phase” (Figure 3). As such, GAPDH is truly the initiator of the “pay-off phase”. The first molecule produced during the “pay-off phase”, NADH, is critically involved in the regulation of intracellular ROS levels, and macromolecular biosynthetic processes. Thus, by producing NADH, GAPDH plays a pivotal role in the cellular redox balance. From a therapeutic point of view given the central role of GAPDH, it is conceivable that, apart from blocking glycolysis and ATP production, GAPDH inhibition would result in multipronged effects within the cancer cell. Inhibition of GAPDH triggers a cascade of events that eventually leads to cancer cell death. First, glucotrioses such as glyceraldehyde-3-phosphate and dihydroxy acetone phosphate accumulate within the cell since they cannot be metabolized. The partial degradation of these glucotrioses results in the formation of a cytotoxic metabolite, methylglyoxal. Normally, the methylglyoxal enters the glyoxalase system to be detoxified. However, in the presence of oxidative stress and GSH depletion secondary to the accumulation of ROS, the glyoxalase 1 (Glo1) activity diminishes leading to the accumulation of methylglyoxal which is directly cytotoxic. Under normal conditions, the methylglyoxal is detoxified by the Glo1 and Glo2 enzymes. But since the activity of both enzymes depends on the level of intracellular GSH, any oxidative stress and resulting increase in ROS level directly and swiftly affect the level of GSH, thus impacting the detoxification ability of methylglyoxal by Glo1 and Glo2 [98]. Inhibition of GAPDH therefore not only directly depletes ATP but also triggers a multipronged attack within the cell. Thus, inhibiting GAPDH not only affects tumor glycolysis (by blocking the most important energy producing step) but also provides an opportunity to exploit other cytotoxic mechanisms related to it.

Since GAPDH represents an attractive target for therapeutic intervention several inhibitors have already been tested for their efficacy in cell cultures as well as animal models [47]. One of the most promising of these inhibitors, the pyruvate analog 3-bromopyruvate (3-BrPA) has demonstrated profound potency in its ability to inhibit tumor glycolysis as well as cause massive depletion of intracellular ATP [99,100]. In addition, 3-BrPA shows utmost specificity and selectivity for GAPDH both *in vitro* in multiple cell lines and *in vivo* in numerous animal models of cancer [101,102]. By binding to GAPDH inside the cancer cells, 3-BrPA depletes ATP profoundly depriving the cancer cells of any energy [103,104]. As a result of its potent anticancer effects, 3-BrPA has recently entered the early phase of clinical trials (phase I).

PK catalyzes the conversion of phosphoenolpyruvate (PEP) to pyruvate, and generates ATP in the process. Among various isoforms, the M2 isoform has gained much attention due to its elevated expression in tumor cells. PK-M2 exists in either active or inactive forms. The activity of this isoform depends on its conformation (tetramer, dimer or monomeric form). PK-M2 is critical for aerobic glycolysis and tumor energy metabolism [49,105]. As with PFK, PK is also regulated by allosteric effectors and by phosphorylation, PK being specifically activated by fructose-1, 6-bisphosphate [106]. Several preclinical studies have shown that PK-M2 could represent a potential therapeutic target [107,108]. Consequently, strategies to develop small molecule inhibitors specific for PK-M2 are in progress. If proven successful in clinical trials, PK-M2 inhibitors could play a significant role in the treatment of cancer [109].

LDH catalyzes the final step in the glycolytic pathway that converts pyruvate into lactate. The intracellular accumulation of lactate is extremely detrimental as the abundance of lactate drastically lowers intracellular pH. The export of lactate into the extracellular space is therefore necessary. It takes place through an active process involving specific transporters known as MCTs. Among the described isoforms, MCT1 and MCT4 have been the subject of intense investigation due to their role in the import and export of lactate. FX11, an inhibitor of LDH depletes intracellular ATP levels, which in turn significantly increase oxidative stress resulting in tumor cell death [110]. Similarly, oxamate, another inhibitor of LDH sensitizes resistant cancer cells to chemotherapeutic agents [111]. Thus inhibition of LDH has demonstrated promising effects in preclinical investigations and its further progress depends on the outcome of clinical trials.

MCTs are the final port of entry for the lactate shuttle. Depending upon the isoform of MCT (1 or 4) the lactate could be either exported or imported [70]. A known MCT inhibitor, α-cyano-4-hydroxy-cinnamic acid, has been shown to affect tumor growth [112]. The principal mechanism underlying the MCT blockade-associated antitumorigenic effects involves intracellular trapping of lactate, which at a prolonged state will result in intracellular acidification causing cancer cell death. However, further studies are warranted to demonstrate the target specificity and therapeutic efficacy of such experimental agents in order to progress towards translation into the clinic.

## Conclusions and future directions

As discussed earlier, the reliance of cancer cells on the glycolytic pathway for their energy needs has been known for decades and has even been successfully exploited diagnostically (FDG-PET imaging) for many years. Yet, targeting this pathway for therapy has not been translated to the clinic. One of the major impediments is to overcome undesirable effects such as systemic toxicity. Plausibly, this is due to the ubiquitous nature of the enzymes of glucose metabolism including glycolysis. But in all fairness, systemic toxicity is a major impediment to clinical progress of any anticancer agent, not just those targeting tumor glycolysis. The emergence of the concept of selective targeting with targeted delivery has provided an additional option to circumvent the problem of systemic toxicity. In the last few years, there has been growing interest in revisiting this approach. This strategy employs the use of image-guidance to deliver the drug where it is required, i.e. in the vicinity of the tumor. Recent advances in imaging technology allow for such precise targeting of tumors, be it directly intra-tumorally or intraarterially where the blood supply to the tumor can be exploited [113]. Such therapeutic approaches provide a unique dual advantage in evading systemic toxicity while improving the potency of the drug [114-116]. However, these approaches are only effective in treating localized disease (to the liver for example) but not for widely metastatic cancers.

Certainly, the scientific rationale for targeting tumor glycolysis is clearly sound and logical. It is based on the fact that tumor glycolysis is a true signature of cancer cells. A number of drug candidates have been tested mostly pre-clinically with mixed success. But some have been extremely promising and are about to enter the clinical arena. One of the keys to clinical success will reside in our ability to develop glycolytic inhibitors with a very high specificity for the molecular target. Recently, Birsoy et al. [117] demonstrated that selective targeting of cancer cells could be achieved if anticancer agents or toxic molecules utilize a mechanism specific for cancer, (such as 3-BrPA that enters cells through MCTs which in turn are upregulated in cancer).

Antiglycolytic agents may provide an additional line of attack in combination therapy. Combining chemotherapeutic drugs and glycolytic inhibitors have already been demonstrated to be promising strategy to overcome drug resistance in cancer. Since tumor glycolysis also plays a significant role in chemoresistance of cancer cells glycolytic inhibitors therefore have the potential to sensitize tumor cells and to improve the outcome of conventional chemotherapy. Such combination therapies have yielded better results in preclinical models. For example, the use of chemotherapeutic agents (adriamycin or paclitaxel) or radiation therapy resulted in improved efficacy, when applied after sensitizing tumor cells with 2-DG, a HKII inhibitor [94,95]. Similarly, several combination studies with the glycolytic inhibitor, 3-BrPA (that primarily targets GAPDH) have demonstrated superior efficacy [118-120]. Thus the combinatorial therapeutic approach remains a viable alternative for treating even resistant phenotypes.

The inhibition of glycolysis can also transform tumor cells into forms that are susceptible or sensitive to immunotherapy, thus opening a new window of opportunity for immunotherapy [121]. In summary, targeting tumor glycolysis is scientifically sound opening the door for a few emerging therapeutic options. Some are about to be tested comprehensively in the clinic. Only then will we know whether the potential exists for the birth of a true viable new class of anti-cancer agents.

## Abbreviations

3-BrPA: 3-bromopyruvate; GLUT: Glucose transporters; HKII: Hexokinase II; GAPDH: Glycerladehyde-3-phosphate dehydrogenase; PFK: Phospho fructo kinase; PFKFB: 6-phosphofructo-2-kinase/fructose-2, 6-bisphosphatases; PK: Pyruvate kinase; LDH: Lactate dehydrogenase; PK-M2: Pyruvate kinase M2; MCT: Monocarboxylate transporters.

## Competing interest

Dr. Geschwind is the founder of Presciencelabs LLC, a biotech firm currently developing the pyruvate analog 3-bromopyruvate (3-BrPA) for clinical use in liver cancer.

## Authors’ contributions

Both authors wrote and approved the manuscript.

## References

[B1] Bomanji JB Costa DC Ell PJ Clinical role of positron emission tomography in oncology Lancet Oncol 2001 2 3 157 164 10.1016/S1470-2045(00)00257-6 11902566

[B2] Warburg O On the origin of cancer cells Science 1956 123 3191 309 314 0036–8075 (Print) 10.1126/science.123.3191.309 13298683

[B3] Warburg O On respiratory impairment in cancer cells Science 1956 124 3215 269 270 PMID- 13471593 OWN - NLM STAT- MEDLINE (0036–8075 (Print)) 13351639

[B4] Geschwind JF Georgiades CS Ko YH Pedersen PL Recently elucidated energy catabolism pathways provide opportunities for novel treatments in hepatocellular carcinoma Expert Rev Anticancer Ther 2004 4 3 449 457 10.1586/14737140.4.3.449 15161443

[B5] Hanahan D Weinberg RA Hallmarks of cancer: the next generation Cell 2011 144 5 646 674 10.1016/j.cell.2011.02.013 21376230

[B6] Macheda ML Rogers S Best JD Molecular and cellular regulation of glucose transporter (GLUT) proteins in cancer J Cell Physiol 2005 202 3 654 662 10.1002/jcp.20166 15389572

[B7] Pelicano H Martin DS Xu RH Huang P Glycolysis inhibition for anticancer treatment Oncogene 2006 25 34 4633 4646 10.1038/sj.onc.1209597 16892078

[B8] Lu CW Lin SC Chen KF Lai YY Tsai SJ Induction of pyruvate dehydrogenase kinase-3 by hypoxia-inducible factor-1 promotes metabolic switch and drug resistance J Biol Chem 2008 283 42 28106 28114 10.1074/jbc.M803508200 18718909 PMC2661383

[B9] Fanciulli M Bruno T Giovannelli A Gentile FP Di Padova M Rubiu O Floridi A Energy metabolism of human LoVo colon carcinoma cells: correlation to drug resistance and influence of lonidamine Clin Cancer Res 2000 6 4 1590 1597 10778993

[B10] Hsu PP Sabatini DM Cancer cell metabolism: Warburg and beyond Cell 2008 134 5 703 707 10.1016/j.cell.2008.08.021 18775299

[B11] Dang CV Hamaker M Sun P Le A Gao P Therapeutic targeting of cancer cell metabolism J Mol Med 2011 89 3 205 212 10.1007/s00109-011-0730-x 21301795 PMC3345191

[B12] Birsoy K Sabatini DM Possemato R Untuning the tumor metabolic machine: targeting cancer metabolism: a bedside lesson Nat Med 2012 18 7 1022 1023 10.1038/nm.2870 22772555

[B13] Dang CV Links between metabolism and cancer Genes Dev 2012 26 9 877 890 10.1101/gad.189365.112 22549953 PMC3347786

[B14] Granchi C Minutolo F Anticancer agents that counteract tumor glycolysis Chem Med Chem 2012 7 8 1318 1350 10.1002/cmdc.201200176 22684868 PMC3516916

[B15] Ward PS Thompson CB Metabolic reprogramming: a cancer hallmark even warburg did not anticipate Cancer Cell 2012 21 3 297 308 10.1016/j.ccr.2012.02.014 22439925 PMC3311998

[B16] Cuperlovic-Culf M Culf AS Touaibia M Lefort N Targeting the latest hallmark of cancer: another attempt at ‘magic bullet’ drugs targeting cancers’ metabolic phenotype Future Oncol 2012 8 10 1315 1330 10.2217/fon.12.121 23130930

[B17] Zhao Y Butler EB Tan M Targeting cellular metabolism to improve cancer therapeutics Cell Death Dis 2013 4 e532 10.1038/cddis.2013.60 23470539 PMC3613838

[B18] Jang M Kim SS Lee J Cancer cell metabolism: implications for therapeutic targets Exp Mol Med 2013 45 e45 10.1038/emm.2013.85 24091747 PMC3809361

[B19] Zhang Y Yang JM Altered energy metabolism in cancer: a unique opportunity for therapeutic intervention Cancer Biol Ther 2013 14 2 81 89 10.4161/cbt.22958 23192270 PMC3572003

[B20] Bonnet S Archer SL Allalunis-Turner J Haromy A Beaulieu C Thompson R Lee CT Lopaschuk GD Puttagunta L Bonnet S Harry G Hashimoto K Porter CJ Andrade MA Thebaud B Michelakis ED A mitochondria-K + channel axis is suppressed in cancer and its normalization promotes apoptosis and inhibits cancer growth Cancer Cell 2007 11 1 37 51 10.1016/j.ccr.2006.10.020 17222789

[B21] Hu Y Lu W Chen G Wang P Chen Z Zhou Y Ogasawara M Trachootham D Feng L Pelicano H Chiao PJ Keating MJ Garcia-Manero G Huang P K-ras(G12V) transformation leads to mitochondrial dysfunction and a metabolic switch from oxidative phosphorylation to glycolysis Cell Res 2012 22 2 399 412 10.1038/cr.2011.145 21876558 PMC3257361

[B22] Lu W Hu Y Chen G Chen Z Zhang H Wang F Feng L Pelicano H Wang H Keating MJ Liu J McKeehan W Wang H Luo Y Huang P Novel role of NOX in supporting aerobic glycolysis in cancer cells with mitochondrial dysfunction and as a potential target for cancer therapy PLoS Biol 2012 10 5 e1001326 10.1371/journal.pbio.1001326 22589701 PMC3348157

[B23] Wallace DC Mitochondria and cancer Nat Rev Cancer 2012 12 10 685 698 10.1038/nrc3365 23001348 PMC4371788

[B24] Cavalli LR Varella-Garcia M Liang BC Diminished tumorigenic phenotype after depletion of mitochondrial DNA Cell Growth Differ 1997 8 11 1189 1198 9372242

[B25] King MP Attardi G Human cells lacking mtDNA: repopulation with exogenous mitochondria by complementation Science 1989 246 4929 500 503 10.1126/science.2814477 2814477

[B26] de Souza AC Justo GZ de Araujo DR Cavagis AD Defining the molecular basis of tumor metabolism: a continuing challenge since Warburg's discovery Cell Physiol Biochem 2011 28 5 771 792 10.1159/000335792 22178931

[B27] Pfeiffer T Schuster S Bonhoeffer S Cooperation and competition in the evolution of ATP-producing pathways Science 2001 292 5516 504 507 10.1126/science.1058079 11283355

[B28] Zhou Y Tozzi F Chen J Fan F Xia L Wang J Gao G Zhang A Xia X Brasher H Widger W Ellis LM Weihua Z Intracellular ATP levels are a pivotal determinant of chemoresistance in colon cancer cells Cancer Res 2012 72 1 304 314 10.1158/0008-5472.CAN-11-1674 22084398 PMC3601736

[B29] Locasale JW Cantley LC Altered metabolism in cancer BMC Biol 2010 8 88 10.1186/1741-7007-8-88 20598111 PMC2892450

[B30] Gatenby RA Gillies RJ Why do cancers have high aerobic glycolysis? Nat Rev Cancer 2004 4 11 891 899 10.1038/nrc1478 15516961

[B31] Lunt SY Vander Heiden MG Aerobic glycolysis: meeting the metabolic requirements of cell proliferation Annu Rev Cell Dev Biol 2011 27 441 464 10.1146/annurev-cellbio-092910-154237 21985671

[B32] Deberardinis RJ Sayed N Ditsworth D Thompson CB Brick by brick: metabolism and tumor cell growth Curr Opin Genet Dev 2008 18 1 54 61 10.1016/j.gde.2008.02.003 18387799 PMC2476215

[B33] Backos DS Franklin CC Reigan P The role of glutathione in brain tumor drug resistance Biochem Pharmacol 2012 83 8 1005 1012 10.1016/j.bcp.2011.11.016 22138445

[B34] Traverso N Ricciarelli R Nitti M Marengo B Furfaro AL Pronzato MA Marinari UM Domenicotti C Role of glutathione in cancer progression and chemoresistance Oxid Med Cell Longev 2013 2013 972913 23766865 10.1155/2013/972913 PMC3673338

[B35] Pitroda SP Wakim BT Sood RF Beveridge MG Beckett MA MacDermed DM Weichselbaum RR Khodarev NN STAT1-dependent expression of energy metabolic pathways links tumour growth and radioresistance to the Warburg effect BMC Med 2009 7 68 10.1186/1741-7015-7-68 19891767 PMC2780454

[B36] Riganti C Gazzano E Polimeni M Aldieri E Ghigo D The pentose phosphate pathway: an antioxidant defense and a crossroad in tumor cell fate Free Radic Biol Med 2012 53 3 421 436 10.1016/j.freeradbiomed.2012.05.006 22580150

[B37] Xu X Zur Hausen A Coy JF Lochelt M Transketolase-like protein 1 (TKTL1) is required for rapid cell growth and full viability of human tumor cells Int J Cancer 2009 124 6 1330 1337 10.1002/ijc.24078 19065656

[B38] Sun W Liu Y Glazer CA Shao C Bhan S Demokan S Zhao M Rudek MA Ha PK Califano JA TKTL1 is activated by promoter hypomethylation and contributes to head and neck squamous cell carcinoma carcinogenesis through increased aerobic glycolysis and HIF1alpha stabilization Clin Cancer Res 2010 16 3 857 866 10.1158/1078-0432.CCR-09-2604 20103683 PMC2824550

[B39] Wanka C Steinbach JP Rieger J Tp53-induced glycolysis and apoptosis regulator (TIGAR) protects glioma cells from starvation-induced cell death by up-regulating respiration and improving cellular redox homeostasis J Biol Chem 2012 287 40 33436 33446 10.1074/jbc.M112.384578 22887998 PMC3460445

[B40] Zhao F Mancuso A Bui TV Tong X Gruber JJ Swider CR Sanchez PV Lum JJ Sayed N Melo JV Perl AE Carroll M Tuttle SW Thompson CB Imatinib resistance associated with BCR-ABL upregulation is dependent on HIF-1alpha-induced metabolic reprograming Oncogene 2010 29 20 2962 2972 10.1038/onc.2010.67 20228846 PMC2874611

[B41] Monteleone F Rosa R Vitale M D'Ambrosio C Succoio M Formisano L Nappi L Romano MF Scaloni A Tortora G Bianco R Zambrano N Increased anaerobic metabolism is a distinctive signature in a colorectal cancer cellular model of resistance to antiepidermal growth factor receptor antibody Proteomics 2013 13 5 866 877 10.1002/pmic.201200303 23281225

[B42] Kim JW Dang CV Multifaceted roles of glycolytic enzymes Trends Biochem Sci 2005 30 3 142 150 10.1016/j.tibs.2005.01.005 15752986

[B43] Pastorino JG Shulga N Hoek JB Mitochondrial binding of hexokinase II inhibits Bax-induced cytochrome c release and apoptosis J Biol Chem 2002 277 9 7610 7618 10.1074/jbc.M109950200 11751859

[B44] Majewski N Nogueira V Bhaskar P Coy PE Skeen JE Gottlob K Chandel NS Thompson CB Robey RB Hay N Hexokinase-mitochondria interaction mediated by Akt is required to inhibit apoptosis in the presence or absence of Bax and Bak Mol Cell 2004 16 5 819 830 10.1016/j.molcel.2004.11.014 15574336

[B45] Seidler NW GAPDH and intermediary metabolism Adv Exp Med Biol 2013 985 37 59 10.1007/978-94-007-4716-6_2 22851446

[B46] Seidler NW Basic biology of GAPDH Adv Exp Med Biol 2013 985 1 36 10.1007/978-94-007-4716-6_1 22851445

[B47] Ganapathy-Kanniappan S Kunjithapatham R Geschwind JF Glyceraldehyde-3-phosphate dehydrogenase: a promising target for molecular therapy in hepatocellular carcinoma Oncotarget 2012 3 9 940 953 22964488 10.18632/oncotarget.623 PMC3660062

[B48] Wu S Le H Dual roles of PKM2 in cancer metabolism Acta Biochim Biophys Sin (Shanghai) 2013 45 1 27 35 10.1093/abbs/gms106 23212076

[B49] Christofk HR Vander Heiden MG Harris MH Ramanathan A Gerszten RE Wei R Fleming MD Schreiber SL Cantley LC The M2 splice isoform of pyruvate kinase is important for cancer metabolism and tumour growth Nature 2008 452 7184 230 233 10.1038/nature06734 18337823

[B50] Luo W Semenza GL Emerging roles of PKM2 in cell metabolism and cancer progression Trends Endocrinol Metab 2012 23 11 560 566 10.1016/j.tem.2012.06.010 22824010 PMC3466350

[B51] Luo W Semenza GL Pyruvate kinase M2 regulates glucose metabolism by functioning as a coactivator for hypoxia-inducible factor 1 in cancer cells Oncotarget 2011 2 7 551 556 21709315 10.18632/oncotarget.299 PMC3248177

[B52] Yang W Zheng Y Xia Y Ji H Chen X Guo F Lyssiotis CA Aldape K Cantley LC Lu Z ERK1/2-dependent phosphorylation and nuclear translocation of PKM2 promotes the Warburg effect Nat Cell Biol 2012 14 12 1295 1304 10.1038/ncb2629 23178880 PMC3511602

[B53] Filipp FV Cancer metabolism meets systems biology: Pyruvate kinase isoform PKM2 is a metabolic master regulator J Carcinog 2013 12 14 10.4103/1477-3163.115423 23961261 PMC3746496

[B54] Yang W Xia Y Hawke D Li X Liang J Xing D Aldape K Hunter T Alfred Yung WK Lu Z PKM2 phosphorylates histone H3 and promotes gene transcription and tumorigenesis Cell 2012 150 4 685 696 10.1016/j.cell.2012.07.018 22901803 PMC3431020

[B55] Yang W Xia Y Ji H Zheng Y Liang J Huang W Gao X Aldape K Lu Z Nuclear PKM2 regulates beta-catenin transactivation upon EGFR activation Nature 2011 480 7375 118 122 22056988 10.1038/nature10598 PMC3235705

[B56] Christofk HR Vander Heiden MG Wu N Asara JM Cantley LC Pyruvate kinase M2 is a phosphotyrosine-binding protein Nature 2008 452 7184 181 186 10.1038/nature06667 18337815

[B57] Diaz-Ruiz R Averet N Araiza D Pinson B Uribe-Carvajal S Devin A Rigoulet M Mitochondrial oxidative phosphorylation is regulated by fructose 1,6-bisphosphate. A possible role in Crabtree effect induction? J Biol Chem 2008 283 40 26948 26955 10.1074/jbc.M800408200 18682403

[B58] Wartenberg M Richter M Datchev A Gunther S Milosevic N Bekhite MM Figulla HR Aran JM Petriz J Sauer H Glycolytic pyruvate regulates P-Glycoprotein expression in multicellular tumor spheroids via modulation of the intracellular redox state J Cell Biochem 2010 109 2 434 446 19950199 10.1002/jcb.22422

[B59] Pertega-Gomes N Vizcaino JR Miranda-Goncalves V Pinheiro C Silva J Pereira H Monteiro P Henrique RM Reis RM Lopes C Baltazar F Monocarboxylate transporter 4 (MCT4) and CD147 overexpression is associated with poor prognosis in prostate cancer BMC Cancer 2011 11 312 10.1186/1471-2407-11-312 21787388 PMC3157459

[B60] Izumi H Takahashi M Uramoto H Nakayama Y Oyama T Wang KY Sasaguri Y Nishizawa S Kohno K Monocarboxylate transporters 1 and 4 are involved in the invasion activity of human lung cancer cells Cancer Sci 2011 102 5 1007 1013 10.1111/j.1349-7006.2011.01908.x 21306479

[B61] Masters C Cellular differentiation and the microcompartmentation of glycolysis Mech Ageing Dev 1991 61 1 11 22 10.1016/0047-6374(91)90003-I 1779698

[B62] Yalcin A Clem BF Simmons A Lane A Nelson K Clem AL Brock E Siow D Wattenberg B Telang S Chesney J Nuclear targeting of 6-phosphofructo-2-kinase (PFKFB3) increases proliferation via cyclin-dependent kinases J Biol Chem 2009 284 36 24223 24232 10.1074/jbc.M109.016816 19473963 PMC2782016

[B63] Demarse NA Ponnusamy S Spicer EK Apohan E Baatz JE Ogretmen B Davies C Direct binding of glyceraldehyde 3-phosphate dehydrogenase to telomeric DNA protects telomeres against chemotherapy-induced rapid degradation J Mol Biol 2009 394 4 789 803 10.1016/j.jmb.2009.09.062 19800890 PMC2789664

[B64] Harada N Yasunaga R Higashimura Y Yamaji R Fujimoto K Moss J Inui H Nakano Y Glyceraldehyde-3-phosphate dehydrogenase enhances transcriptional activity of androgen receptor in prostate cancer cells J Biol Chem 2007 282 31 22651 22661 10.1074/jbc.M610724200 17553795

[B65] Popanda O Fox G Thielmann HW Modulation of DNA polymerases alpha, delta and epsilon by lactate dehydrogenase and 3-phosphoglycerate kinase Biochim Biophys Acta 1998 1397 1 102 117 10.1016/S0167-4781(97)00229-7 9545551

[B66] Walenta S Wetterling M Lehrke M Schwickert G Sundfor K Rofstad EK Mueller-Klieser W High lactate levels predict likelihood of metastases, tumor recurrence, and restricted patient survival in human cervical cancers Cancer Res 2000 60 4 916 921 10706105

[B67] Sonveaux P Vegran F Schroeder T Wergin MC Verrax J Rabbani ZN De Saedeleer CJ Kennedy KM Diepart C Jordan BF Kelley MJ Gallez B Wahl ML Feron O Dewhirst MW Targeting lactate-fueled respiration selectively kills hypoxic tumor cells in mice J Clin Invest 2008 118 12 3930 3942 19033663 10.1172/JCI36843 PMC2582933

[B68] Draoui N Feron O Lactate shuttles at a glance: from physiological paradigms to anti-cancer treatments Dis Model Mech 2011 4 6 727 732 10.1242/dmm.007724 22065843 PMC3209642

[B69] Semenza GL Tumor metabolism: cancer cells give and take lactate J Clin Invest 2008 118 12 3835 3837 19033652 10.1172/JCI37373 PMC2582934

[B70] Pinheiro C Longatto-Filho A Azevedo-Silva J Casal M Schmitt FC Baltazar F Role of monocarboxylate transporters in human cancers: state of the art J Bioenerg Biomembr 2012 44 1 127 139 10.1007/s10863-012-9428-1 22407107

[B71] Feron O Pyruvate into lactate and back: from the Warburg effect to symbiotic energy fuel exchange in cancer cells Radiother Oncol 2009 92 3 329 333 10.1016/j.radonc.2009.06.025 19604589

[B72] Hussien R Brooks GA Mitochondrial and plasma membrane lactate transporter and lactate dehydrogenase isoform expression in breast cancer cell lines Physiol Genomics 2011 43 5 255 264 10.1152/physiolgenomics.00177.2010 21177384 PMC3068517

[B73] Hugo-Wissemann D Anundi I Lauchart W Viebahn R de Groot H Differences in glycolytic capacity and hypoxia tolerance between hepatoma cells and hepatocytes Hepatology 1991 13 2 297 303 10.1002/hep.1840130215 1847350

[B74] Mikuriya K Kuramitsu Y Ryozawa S Fujimoto M Mori S Oka M Hamano K Okita K Sakaida I Nakamura K Expression of glycolytic enzymes is increased in pancreatic cancerous tissues as evidenced by proteomic profiling by two-dimensional electrophoresis and liquid chromatography-mass spectrometry/mass spectrometry Int J Oncol 2007 30 4 849 855 17332923

[B75] Yeh CS Wang JY Chung FY Lee SC Huang MY Kuo CW Yang MJ Lin SR Significance of the glycolytic pathway and glycolysis related-genes in tumorigenesis of human colorectal cancers Oncol Rep 2008 19 1 81 91 18097579

[B76] Rimpi S Nilsson JA Metabolic enzymes regulated by the Myc oncogene are possible targets for chemotherapy or chemoprevention Biochem Soc Trans 2007 35 Pt 2 305 310 17371266 10.1042/BST0350305

[B77] Robey IF Lien AD Welsh SJ Baggett BK Gillies RJ Hypoxia-inducible factor-1alpha and the glycolytic phenotype in tumors Neoplasia 2005 7 4 324 330 10.1593/neo.04430 15967109 PMC1501147

[B78] Rodriguez-Enriquez S Gallardo-Perez JC Aviles-Salas A Marin-Hernandez A Carreno-Fuentes L Maldonado-Lagunas V Moreno-Sanchez R Energy metabolism transition in multi-cellular human tumor spheroids J Cell Physiol 2008 216 1 189 197 10.1002/jcp.21392 18264981

[B79] Vivanco I Sawyers CL The phosphatidylinositol 3-Kinase AKT pathway in human cancer Nat Rev Cancer 2002 2 7 489 501 10.1038/nrc839 12094235

[B80] Elstrom RL Bauer DE Buzzai M Karnauskas R Harris MH Plas DR Zhuang H Cinalli RM Alavi A Rudin CM Thompson CB Akt stimulates aerobic glycolysis in cancer cells Cancer Res 2004 64 11 3892 3899 10.1158/0008-5472.CAN-03-2904 15172999

[B81] Csibi A Blenis J Appetite for destruction: the inhibition of glycolysis as a therapy for tuberous sclerosis complex-related tumors BMC Biol 2011 9 69 10.1186/1741-7007-9-69 22018140 PMC3198763

[B82] Jiang X Kenerson H Aicher L Miyaoka R Eary J Bissler J Yeung RS The tuberous sclerosis complex regulates trafficking of glucose transporters and glucose uptake Am J Pathol 2008 172 6 1748 1756 10.2353/ajpath.2008.070958 18511518 PMC2408433

[B83] Duvel K Yecies JL Menon S Raman P Lipovsky AI Souza AL Triantafellow E Ma Q Gorski R Cleaver S Vander Heiden MG MacKeigan JP Finan PM Clish CB Murphy LO Manning BD Activation of a metabolic gene regulatory network downstream of mTOR complex 1 Mol Cell 2010 39 2 171 183 10.1016/j.molcel.2010.06.022 20670887 PMC2946786

[B84] Klement RJ Kammerer U Is there a role for carbohydrate restriction in the treatment and prevention of cancer? Nutr Metab (Lond) 2011 8 75 10.1186/1743-7075-8-75 22029671 PMC3267662

[B85] Liu Y Cao Y Zhang W Bergmeier S Qian Y Akbar H Colvin R Ding J Tong L Wu S Hines J Chen X A small-molecule inhibitor of glucose transporter 1 downregulates glycolysis, induces cell-cycle arrest, and inhibits cancer cell growth in vitro and in vivo Mol Cancer Ther 2012 11 8 1672 1682 10.1158/1535-7163.MCT-12-0131 22689530

[B86] Mathupala SP Ko YH Pedersen PL Hexokinase II: cancer's double-edged sword acting as both facilitator and gatekeeper of malignancy when bound to mitochondria Oncogene 2006 25 34 4777 4786 10.1038/sj.onc.1209603 16892090 PMC3385868

[B87] Mathupala SP Ko YH Pedersen PL Hexokinase-2 bound to mitochondria: cancer’s stygian link to the “Warburg Effect” and a pivotal target for effective therapy Semin Cancer Biol 2009 19 1 17 24 10.1016/j.semcancer.2008.11.006 19101634 PMC2714668

[B88] Price GS Page RL Riviere JE Cline JM Thrall DE Pharmacokinetics and toxicity of oral and intravenous lonidamine in dogs Cancer Chemother Pharmacol 1996 38 2 129 135 10.1007/s002800050460 8616902

[B89] Maher JC Krishan A Lampidis TJ Greater cell cycle inhibition and cytotoxicity induced by 2-deoxy-D-glucose in tumor cells treated under hypoxic vs aerobic conditions Cancer Chemother Pharmacol 2004 53 2 116 122 10.1007/s00280-003-0724-7 14605866

[B90] Kurtoglu M Gao N Shang J Maher JC Lehrman MA Wangpaichitr M Savaraj N Lane AN Lampidis TJ Under normoxia, 2-deoxy-D-glucose elicits cell death in select tumor types not by inhibition of glycolysis but by interfering with N-linked glycosylation Mol Cancer Ther 2007 6 11 3049 3058 10.1158/1535-7163.MCT-07-0310 18025288

[B91] Zhong D Liu X Schafer-Hales K Marcus AI Khuri FR Sun SY Zhou W 2-Deoxyglucose induces Akt phosphorylation via a mechanism independent of LKB1/AMP-activated protein kinase signaling activation or glycolysis inhibition Mol Cancer Ther 2008 7 4 809 817 10.1158/1535-7163.MCT-07-0559 18413794

[B92] Zhong D Xiong L Liu T Liu X Liu X Chen J Sun SY Khuri FR Zong Y Zhou Q Zhou W The glycolytic inhibitor 2-deoxyglucose activates multiple prosurvival pathways through IGF1R J Biol Chem 2009 284 35 23225 23233 10.1074/jbc.M109.005280 19574224 PMC2749096

[B93] Maher JC Wangpaichitr M Savaraj N Kurtoglu M Lampidis TJ Hypoxia-inducible factor-1 confers resistance to the glycolytic inhibitor 2-deoxy-D-glucose Mol Cancer Ther 2007 6 2 732 741 10.1158/1535-7163.MCT-06-0407 17308069

[B94] Maschek G Savaraj N Priebe W Braunschweiger P Hamilton K Tidmarsh GF De Young LR Lampidis TJ 2-deoxy-D-glucose increases the efficacy of adriamycin and paclitaxel in human osteosarcoma and non-small cell lung cancers in vivo Cancer Res 2004 64 1 31 34 10.1158/0008-5472.CAN-03-3294 14729604

[B95] Dwarakanath B Jain V Targeting glucose metabolism with 2-deoxy-D-glucose for improving cancer therapy Future Oncol 2009 5 5 581 585 10.2217/fon.09.44 19519197

[B96] Chesney J 6-Phosphofructo-2-Kinase/fructose-2,6-Bisphosphatase and Tumor Cell Glycolysis Curr Opin Clin Nutr Metab Care 2006 9 5 535 539 10.1097/01.mco.0000241661.15514.fb 16912547

[B97] Clem B Telang S Clem A Yalcin A Meier J Simmons A Rasku MA Arumugam S Dean WL Eaton J Lane A Trent JO Chesney J Small-molecule inhibition of 6-phosphofructo-2-kinase activity suppresses glycolytic flux and tumor growth Mol Cancer Ther 2008 7 1 110 120 10.1158/1535-7163.MCT-07-0482 18202014

[B98] Thornalley PJ Rabbani N Glyoxalase in tumourigenesis and multidrug resistance Semin Cell Dev Biol 2011 22 3 318 325 10.1016/j.semcdb.2011.02.006 21315826

[B99] Ko YH Smith BL Wang Y Pomper MG Rini DA Torbenson MS Hullihen J Pedersen PL Advanced cancers: eradication in all cases using 3-bromopyruvate therapy to deplete ATP Biochem Biophys Res Commun 2004 324 1 269 275 10.1016/j.bbrc.2004.09.047 15465013

[B100] Pedersen PL The cancer cell's “power plants” as promising therapeutic targets: an overview J Bioenerg Biomembr 2007 39 1 1 12 10.1007/s10863-007-9070-5 17404823

[B101] Ganapathy-Kanniappan S Vali M Kunjithapatham R Buijs M Syed LH Rao PP Ota S Kwak BK Loffroy R Geschwind JF 3-Bromopyruvate: a New Targeted Antiglycolytic Agent and a Promise for Cancer Therapy Curr Pharm Biotechnol 2010 11 5 510 517 10.2174/138920110791591427 20420565

[B102] Ganapathy-Kanniappan S Kunjithapatham R Geschwind JF Anticancer efficacy of the metabolic blocker 3-bromopyruvate: specific molecular targeting Anticancer Res 2013 33 1 13 20 23267123

[B103] Ganapathy-Kanniappan S Geschwind JF Kunjithapatham R Buijs M Vossen JA Tchernyshyov I Cole RN Syed LH Rao PP Ota S Vali M Glyceraldehyde-3-phosphate dehydrogenase (GAPDH) is pyruvylated during 3-bromopyruvate mediated cancer cell death Anticancer Res 2009 29 12 4909 4918 20044597 PMC3743725

[B104] Pereira da Silva AP El-Bacha T Kyaw N dos Santos RS da-Silva WS Almeida FC Da Poian AT Galina A Inhibition of energy-producing pathways of HepG2 cells by 3-bromopyruvate Biochem J 2009 417 3 717 726 10.1042/BJ20080805 18945211

[B105] Spoden GA Mazurek S Morandell D Bacher N Ausserlechner MJ Jansen-Durr P Eigenbrodt E Zwerschke W Isotype-specific inhibitors of the glycolytic key regulator pyruvate kinase subtype M2 moderately decelerate tumor cell proliferation Int J Cancer 2008 123 2 312 321 10.1002/ijc.23512 18425820

[B106] Dombrauckas JD Santarsiero BD Mesecar AD Structural basis for tumor pyruvate kinase M2 allosteric regulation and catalysis Biochemistry 2005 44 27 9417 9429 10.1021/bi0474923 15996096

[B107] Chen J Xie J Jiang Z Wang B Wang Y Hu X Shikonin and its analogs inhibit cancer cell glycolysis by targeting tumor pyruvate kinase-M2 Oncogene 2011 30 42 4297 4306 10.1038/onc.2011.137 21516121

[B108] Goldberg MS Sharp PA Pyruvate kinase M2-specific siRNA induces apoptosis and tumor regression J Exp Med 2012 209 2 217 224 10.1084/jem.20111487 22271574 PMC3280873

[B109] Vander Heiden MG Christofk HR Schuman E Subtelny AO Sharfi H Harlow EE Xian J Cantley LC Identification of small molecule inhibitors of pyruvate kinase M2 Biochem Pharmacol 2010 79 8 1118 1124 10.1016/j.bcp.2009.12.003 20005212 PMC2823991

[B110] Le A Cooper CR Gouw AM Dinavahi R Maitra A Deck LM Royer RE Vander Jagt DL Semenza GL Dang CV Inhibition of lactate dehydrogenase A induces oxidative stress and inhibits tumor progression Proc Natl Acad Sci U S A 2010 107 5 2037 2042 10.1073/pnas.0914433107 20133848 PMC2836706

[B111] Zhou M Zhao Y Ding Y Liu H Liu Z Fodstad O Riker AI Kamarajugadda S Lu J Owen LB Ledoux SP Tan M Warburg effect in chemosensitivity: targeting lactate dehydrogenase-A re-sensitizes taxol-resistant cancer cells to taxol Mol Cancer 2010 9 33 20144215 10.1186/1476-4598-9-33 PMC2829492

[B112] Colen CB Shen Y Ghoddoussi F Yu P Francis TB Koch BJ Monterey MD Galloway MP Sloan AE Mathupala SP Metabolic targeting of lactate efflux by malignant glioma inhibits invasiveness and induces necrosis: an in vivo study Neoplasia 2011 13 7 620 632 21750656 10.1593/neo.11134 PMC3132848

[B113] Lencioni R Loco-regional treatment of hepatocellular carcinoma Hepatology 2010 52 2 762 773 10.1002/hep.23725 20564355

[B114] Geschwind JF Ko YH Torbenson MS Magee C Pedersen PL Novel therapy for liver cancer: direct intraarterial injection of a potent inhibitor of ATP production Cancer Res 2002 62 14 3909 3913 12124317

[B115] Vali M Liapi E Kowalski J Hong K Khwaja A Torbenson MS Georgiades C Geschwind JF Intraarterial therapy with a new potent inhibitor of tumor metabolism (3-bromopyruvate): identification of therapeutic dose and method of injection in an animal model of liver cancer J Vasc Interv Radiol 2007 18 1 Pt 1 95 101 17296709 10.1016/j.jvir.2006.10.019

[B116] Vali M Vossen JA Buijs M Engles JM Liapi E Ventura VP Khwaja A Acha-Ngwodo O Shanmugasundaram G Syed L Wahl RL Geschwind JF Targeting of VX2 rabbit liver tumor by selective delivery of 3-bromopyruvate: a biodistribution and survival study J Pharmacol Exp Ther 2008 327 1 32 37 10.1124/jpet.108.141093 18591216 PMC2760588

[B117] Birsoy K Wang T Possemato R Yilmaz OH Koch CE Chen WW Hutchins AW Gultekin Y Peterson TR Carette JE Brummelkamp TR Clish CB Sabatini DM MCT1-mediated transport of a toxic molecule is an effective strategy for targeting glycolytic tumors Nat Genet 2013 45 1 104 108 23202129 10.1038/ng.2471 PMC3530647

[B118] Cao X Bloomston M Zhang T Frankel WL Jia G Wang B Hall NC Koch RM Cheng H Knopp MV Sun D Synergistic antipancreatic tumor effect by simultaneously targeting hypoxic cancer cells with HSP90 inhibitor and glycolysis inhibitor Clin Cancer Res 2008 14 6 1831 1839 10.1158/1078-0432.CCR-07-1607 18347186

[B119] Filomeni G Cardaci S Da Costa Ferreira AM Rotilio G Ciriolo MR Metabolic oxidative stress elicited by the copper(II) complex [Cu(isaepy)2] triggers apoptosis in SH-SY5Y cells through the induction of the AMP-activated protein kinase/p38MAPK/p53 signalling axis: evidence for a combined use with 3-bromopyruvate in neuroblastoma treatment Biochem J 2011 437 3 443 453 10.1042/BJ20110510 21548882

[B120] Nakano A Tsuji D Miki H Cui Q El Sayed SM Ikegame A Oda A Amou H Nakamura S Harada T Fujii S Kagawa K Takeuchi K Sakai A Ozaki S Okano K Nakamura T Itoh K Matsumoto T Abe M Glycolysis inhibition inactivates ABC transporters to restore drug sensitivity in malignant cells PLoS One 2011 6 11 e27222 10.1371/journal.pone.0027222 22073292 PMC3206937

[B121] Beneteau M Zunino B Jacquin MA Meynet O Chiche J Pradelli LA Marchetti S Cornille A Carles M Ricci JE Combination of glycolysis inhibition with chemotherapy results in an antitumor immune response Proc Natl Acad Sci U S A 2012 109 49 20071 20076 10.1073/pnas.1206360109 23169636 PMC3523878

